# Expression of Interleukin-6 and the Interleukin-6 Receptor Predicts the Clinical Outcomes of Patients with Soft Tissue Sarcomas

**DOI:** 10.3390/cancers12030585

**Published:** 2020-03-03

**Authors:** Koichi Nakamura, Tomoki Nakamura, Takahiro Iino, Tomohito Hagi, Kouji Kita, Kunihiro Asanuma, Akihiro Sudo

**Affiliations:** Department of Orthopaedic Surgery, Mie University Graduate School of Medicine, Tsu 514-8507, Japan; k-nakamura@clin.medic.mie-u.ac.jp (K.N.); tiino@clin.medic.mie-u.ac.jp (T.I.); hagifana@clin.medic.mie-u.ac.jp (T.H.); kkita@clin.medic.mie-u.ac.jp (K.K.); kasanum@clin.medic.mie-u.ac.jp (K.A.); a-sudou@clin.medic.mie-u.ac.jp (A.S.)

**Keywords:** soft tissue sarcoma, interleukin-6, interleukin-6 receptor, tissue expression, prognosis

## Abstract

Interleukin-6 (IL-6) affects the key parameters of oncogenesis, which increases the cell resistance to apoptosis, the proliferation of cancer cells, angiogenesis, invasion, malignancy, and the ability of tumor cells to respond to anticancer therapy. This study aimed to elucidate the association between IL-6 and IL-6 receptor (IL-6R) expression in tissues and clinical outcomes in patients with soft tissue sarcomas (STSs) because, to our knowledge, this has not been done before. We enrolled 86 patients with histologically-proven localized STSs who underwent surgical resection. The cohort included 48 men and 38 women, with a mean age of 65.6 years. The mean follow-up duration was 40.5 months. The expression of IL-6 and IL-6R was immunohistochemically determined. We analyzed prognostic factors for overall survival (OS) and metastasis-free survival (MFS). High IL-6 expression was observed in 23.3% (20/86), high IL-6R expression in 44.2% (38/86), and high expression of both in 16.3% (14/86) of patients. Multivariate analysis showed that a high expression of both IL-6 and IL-6R was a prognostic factor for OS and MFS. We found that this high expression indicated that the patient had a poor prognosis for OS and MFS.

## 1. Introduction

The inflammatory microenvironment plays an important role in the development of cancer [1]. Interleukin-6 (IL-6) is an essential cytokine in the cytokine cascade involved in the generation and regulation of inflammation [2]. Serum IL-6 levels have been reported to be associated with the prognosis of and tolerance to chemotherapy in several types of cancer, as well as with early recurrence [3,4]. A previous study demonstrated that the presence of systemic inflammation was a prognostic factor for patients with soft tissue sarcomas (STSs) [5,6,7]. Moreover, elevated levels of serum IL-6 were identified as important factors for survival and event-free survival in patients with STSs [2,4,8].

Previously, we reported a relationship between the levels of serum IL-6 and its diagnostic and prognostic value in patients with STSs [9]. Elevated serum IL-6 levels are a predictor of STSs, and C-reactive protein (CRP) levels are strongly correlated with serum IL-6 levels [9]. We suggested that the measurement of IL-6 levels may be a useful method for identifying patients who are at a high risk of STSs and tumor-related death [9].

In addition to serum inflammatory biomarkers, the expression of inflammatory cytokines produced by tumor cells has been found to be related to survival in several cancer types. IL-6 expression in tissues was significantly associated with a poor prognosis in patients with squamous cell carcinoma of the esophagus [10]. IL-6 expression in tissues was significantly associated with invasion depth and lymph node metastasis in colorectal cancer; it also correlated with several clinicopathological factors, such as TMN stage [11]. Furthermore, IL-6 receptor (IL-6R) expression has been reported in cancer cells. IL-6R expression in tissues has been reported to be significantly associated with invasion depth and lymph node metastasis in gastric cancer [12]. Moreover, IL-6R in tissues has been found to be a prognostic factor for survival in cervical cancer [13,14].

However, to the best of our knowledge, no previous studies have evaluated the association between IL-6 and IL-6R expression in tissues and clinical outcomes in patients with STSs. Therefore, this was the aim of the present study.

## 2. Results

### 2.1. Patient, Tumor, and Treatment Characteristics (Table 1)

In total, 86 patients were included in the study. They were histologically classified as follows: well-differentiated liposarcoma (*n* = 22), undifferentiated pleomorphic sarcoma (*n* = 13), myxofibrosarcoma (*n* = 14), dedifferentiated liposarcoma (*n* = 14), leiomyosarcoma (*n* = 9), malignant peripheral nerve sheath tumor (*n* = 3), myxoid liposarcoma (*n* = 3), synovial sarcoma (*n* = 4), and other high-grade sarcomas (*n* = 4).

According to the French Federation of Cancer Centers Sarcoma Group (FNCLCC) histological grading system, 25 STSs were grade 1, 27 were grade 2, and 34 were grade 3. All patients underwent surgical tumor resection. Adjuvant radiotherapy was performed in 21 patients. Adjuvant or neoadjuvant chemotherapy was administered to 19 patients. Of 19 patients, 12 patients received neo-adjuvant chemotherapy.

### 2.2. Expression of IL-6 and IL-6R in the Tumor Tissues of Patients with STSs

A high expression of IL-6 was observed in 23.3% (20/86) of cases; thus, a low expression was observed in 76.7% (66/86). The patients exhibiting a high expression of IL-6 were significantly older than those exhibiting a low expression (*p* = 0.03). Of the 61 patients with high-grade STSs, a high expression of IL-6 was observed in 29.5% (18/61), while a high expression of IL-6 was observed in 8.0% (2/25) of patients with low-grade STSs (*p* = 0.047). The mean serum IL-6 level was 90.3 pg/mL (median 31.6 pg/mL) in patients with STSs exhibiting high IL-6 expression and 12.5 pg/mL (median 5.3 pg/mL) in patients with STSs exhibiting low IL-6 expression (*p* = 0.001). 

A high expression of IL-6R was observed in 44.2% (38/86) of patients, whereas a low expression was observed in 55.8% (48/86). There were no differences in patient age between those exhibiting a high or low expression of IL-6R (*p* = 0.98). A high expression of IL-6R was observed in 54.1% (33/61) of patients with high-grade STSs and in 20.0% (5/25) of those with low-grade STSs (*p* = 0.004). The mean IL-6 level was 38.9 pg/mL (median 9.17 pg/mL) in patients exhibiting a high expression of IL-6R and 24.0 pg/mL (median 6.10 pg/mL) in those exhibiting a low expression (*p* = 0.36). 

A high expression of both IL-6 and IL-6R in tissues was observed in 16.3% (14/86) of patients. Tumor grade, serum CRP, and IL-6 levels in patients exhibiting a high expression of both IL-6 and IL-6R were high (Table 2).

### 2.3. Overall Survival (OS), Metastasis-Free Survival (MFS), and Prognostic Factors

As of August 2019, 45 of the 86 patients had a continuous disease-free status, 6 had no evidence of disease, 10 were alive with the disease, 20 had died of the disease, and 5 had died of other causes. The five-year OS rate was 66.1% (95% CI: 51.5–77.2%). The five-year MFS rate was 59.8% (95% CI: 46.9–70.6%). 

Patients exhibiting high IL-6 expression in tissues had poorer OS results than those exhibiting low expression. The OS at 5 years in patients exhibiting a high and low expression of IL-6 was 48.7% (95% CI: 25.7–68.4%) and 72.0% (95% CI: 52.6–84.5%), respectively (*p* = 0.01). The MFS at 5 years in patients exhibiting a high and low expression of IL-6 was 40.0% (95% CI: 19.3–60.0%) and 65.2% (95% CI: 48.7–77.5%), respectively (*p* < 0.01). Next, we examined the OS, MFS, and prognostic factors of patients exhibiting a high and low expression of IL-6R. The OS at 5 years in patients exhibiting a high and low expression of IL-6R was 57.2% (95% CI: 37.2–72.9%) and 74.7% (95% CI: 52.9–87.5%), respectively (*p* = 0.03). The MFS at 5 years in patients exhibiting a high and low expression of IL-6R was 40.7% (95% CI: 23.6–57.2%) and 77.0% (95% CI: 59.8–87.5%), respectively (*p* < 0.001). 

**Figure 1:**
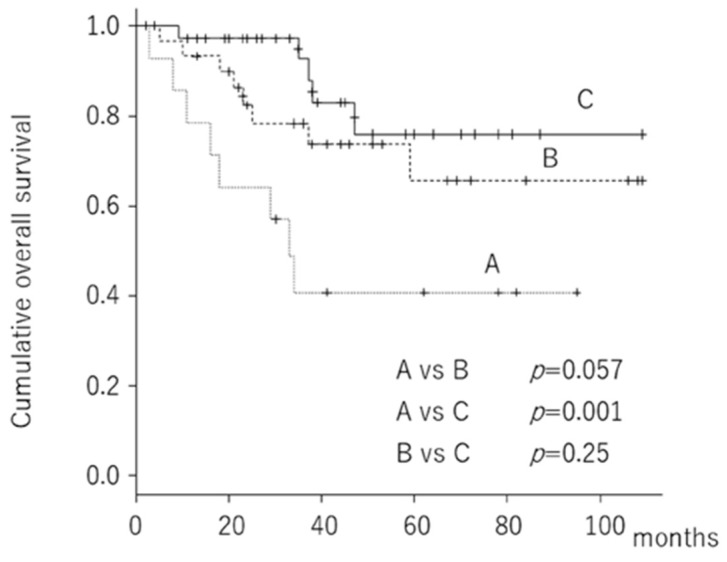
Kaplan–Meier curve showing overall survival according to the expression of IL-6 and IL-6R in the tumor tissue.

Finally, we divided the 86 patients into three groups according to the expression of IL-6 and IL-6R in the tissues: (a) high expression of both IL-6 and IL-6R, (b) high expression of IL-6 or IL-6R, and (c) low expression of both IL-6 and IL-6R. The OS at 5 years was 40.8% (95% CI: 15.6–64.9%) for patients exhibiting a high expression of both IL-6 and IL-6R, 65.6% (95% CI: 40.1–82.3%) for patients exhibiting a high expression of either IL-6 or IL-6R, and 76.1% (95% CI: 50.0–89.8%) for patients exhibiting a low expression of both IL-6 and IL-6R (Figure 1).

Univariate Cox hazard analysis revealed that a high expression of both IL-6 and IL-6R in tissues was a poorer prognostic factor for predicting OS than a low expression of both IL-6 and IL-6R. Age and tumor grade were also related to survival (Table 3). Upon multivariate Cox population hazard analysis, a high expression of both IL-6 and IL-6R in tissues and age remained prognostic factors for predicting OS (Table 4).

**Figure 2:**
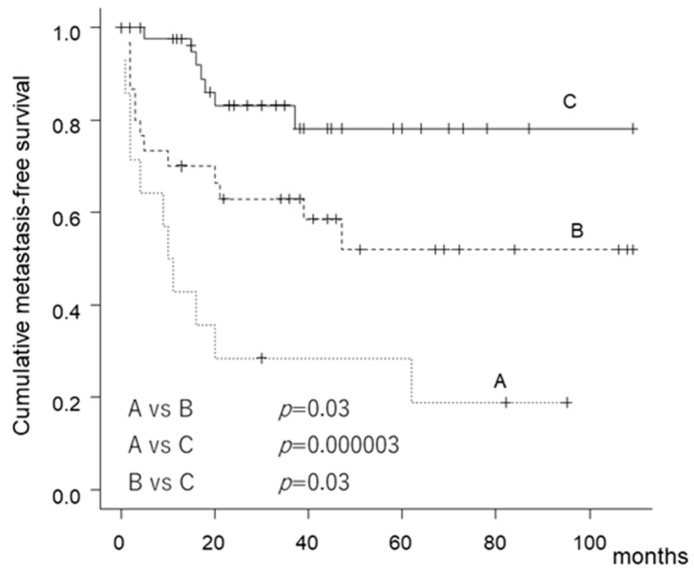
Kaplan–Meier curve showing metastasis-free survival according to the expression of IL-6 and IL-6R in the tumor tissue.

Patients exhibiting a high expression of both IL-6 and IL-6R had poor MFS. The MFS at 5 years was 28.6% (95% CI: 8.8–52.4%) for patients exhibiting a high expression of both IL-6 and IL-6R, 52% (95% CI: 30.6–69.7%) for patients exhibiting a high expression of either IL-6 or IL-6R, and 78.2% (95% CI: 58.6–89.3%) for patients exhibiting a low expression of both IL-6 and IL-6R (Figure 2).

Univariate Cox hazard analysis revealed that patients exhibiting a high expression of both IL-6 and IL-6R had poorer MFS than those exhibiting a low expression. Patients exhibiting a high expression of either IL-6 or IL-6R in tissues had poorer MFS than those exhibiting a low expression (Table 3). Age was also a prognostic factor for predicting MFS. With regard to the multivariate analysis, a high expression of both IL-6 and IL-6R, a high expression of either IL-6 or IL-6R, and age were prognostic factors for predicting MFS (Table 5). Finally, we statistically validated the power of the study. The power for OS and MFS was 86% and 99%, respectively.

## 3. Discussion

IL-6 has pleiotropic effects on various cell types in the tumor microenvironment, which leads to the regulation of pro-oncogenic transcription factors NF-κB and STAT3 [15,16]. IL-6 affects the key parameters of oncogenesis, which increases cell resistance to apoptosis, the proliferation of cancer cells, angiogenesis, invasion, malignancy, and the ability of tumor cells to respond to anticancer therapies [15]. Serum IL-6 levels have been reported to be associated with the prognosis of several tumors. A systematic review reported that serum IL-6 levels were increased in the majority of clinical cancer studies, with a significant correlation between serum IL-6 levels and survival being documented in 86% of patients in 23 different cancer types [2].

In the present study, we showed the relationship between the expression of IL-6 and IL-6R in tissues and survival and MFS in patients with STSs. We also demonstrated the relationship between serum IL-6 and CRP levels and the expression of IL-6 in tissues. Kinoshita et al. reported that the levels of IL-6 in the serum reflected the levels in the tumor component [4]. Autocrine loops of IL-6 and IL-6R exist in several tumors [16,17]. IL-6 was found to be secreted by renal cancer cells to act as an autocrine tumor growth factor to induce the transcriptional inflammatory response and promote tumor progression through the JAK-STAT pathway [18]. Although there have been many studies on the relationship between serum IL-6 and clinical outcomes in various cancers, there have been few studies investigating the relationship between IL-6 and IL-6R in tissues and clinical outcomes. 

In the present study, we showed that a high expression of both IL-6 and IL-6R in tissues was related to MFS and OS in patients with STSs. Fu et al. reported that the positive expression of IL-6 and IL-6R in renal cell cancer was significantly associated with poor survival in multivariate analysis [19]. They also found that patients with a positive expression of both IL-6 and IL-6R had shorter cancer-specific survival than other groups (IL-6 negative/IL-6R positive, IL-6 positive/IL-6R negative, and IL-6 negative/IL-6R negative); these results support those of the present study. Interestingly, we found that patients exhibiting a high expression of either IL-6 or IL-6R had poorer MFS than those exhibiting a low expression of both IL-6 and IL-6R. Labovsky et al. reported that the expression of IL-6R in spindle stromal cells, such as carcinoma-associated fibroblasts and mesenchymal stem cells, was associated with disease-free survival (MFS) in patients with early breast cancer. Although we did not investigate these factors, tumor circumstance and cells may have affected the IL-6 pathway [20]. IL-6 and IL-6R may be potential therapeutic targets. The monoclonal anti-IL-6 antibody siltuximab showed anti-tumor effects against prostate cancer, renal cell cancer, and multiple myeloma [21]. The clinical trials using humanized anti-IL6R antibody tocilizumab are also approved for the on-label use of tocilizumab [21]. We believe that STS patients with a high expression of IL-6 and IL-6R may also be potential candidates for those clinical trials because their prognosis may be poor.

There are several limitations in the present study, including the retrospective design and the inclusion of 12 patients who received neo-adjuvant chemotherapy. The response to neoadjuvant chemotherapy may have affected the expression of IL-6 and IL6R. No correction for multiple testing was performed. However, this study is the first, to our knowledge, to describe the relationship between IL-6 and IL-6R in tissues and clinical outcomes in patients with STSs. However, further studies are necessary to validate this. We also tried to quantify the IL-6 and IL-6R levels using real- time PCR, but the tumor sample was not large enough to evaluate them.

In conclusion, we found that patients exhibiting a high expression of both IL-6 and IL-6R in tissues had a poor OS and MFS prognosis. We believe that the IL-6 autocrine loop in patients with STSs may be associated with tumor progression.

## 4. Materials and Methods

### 4.1. Patients and Methods

The study was approved (No.2804) by the Ethics Committee of Mie University Hospital. Written informed consent was obtained from all patients. The design and procedures of the study were carried out in accordance with the principles of the Declaration of Helsinki.

In total, 86 patients with STSs who underwent surgical resection between December 2008 and December 2017 at Mie University Hospital were retrospectively reviewed. Although we also treated another 30 patients with STSs, they were excluded from the present study because of a lack of information or disagreement of the study. The cohort included 48 men and 38 women, with a mean age of 65.6 years (range 10–88 years) at first presentation (Table 1). The mean follow-up duration was 40.5 months (range 1–109 months). Twenty patients who presented with recurrent disease and/or metastasis and 27 patients who were referred for additional resection after a previous inadequate excision were excluded, respectively. Four patients with an obvious history of myocardial infarction or infectious disease were also excluded. Histopathological diagnosis and tumor grade, determined using the French Federation of Cancer Centers Sarcoma Group (FNCLCC) system [22], were reviewed in all patients and confirmed by independent pathologists. We classified FNCLCC Grade 1 tumors as low-grade and Grade 2 and 3 tumors as high-grade. Blood samples from all patients were obtained within 1 month before initial treatment. All blood samples were stored at −80 °C until analysis. Blood samples were centrifuged at 1000× *g* for 15 min. The levels of IL-6 were measured using an R&D Systems™ Quantikine^®^ ELISA kit (R&D System Inc., Minneapolis, MN, USA). 

### 4.2. Immunohistochemical Staining

Tumor tissue sections were subjected to immunohistochemistry analysis. Tumor blocks were formalin-fixed, paraffin-embedded, and sliced into 4 μm-thick sections. These were deparaffinized in xylene and rehydrated using diminishing concentrations of ethanol (100%, 95%, 85%, and 75%). This was followed by subsequent incubation in 3% H_2_O_2_ for 30 min in the dark at room temperature to eliminate endogenous peroxidase activity. Antigen retrieval was performed by heating the sections for 10 min in citrate buffer (pH 6.0) using the autoclave sterilizer method. The sections were allowed to cool at room temperature for 60 min and then rinsed three times for 5 min with fresh PBS. Thereafter, the slides were preincubated with healthy bovine serum albumin diluted in PBS (pH 7.4) for 15 min at 37 °C and then incubated overnight at room temperature with primary antibodies specific for IL-6 (rabbit anti-IL-6, dilution 1:30, Santa Cruz Biotechnology, Dallas, TX, USA) and IL-6R (rabbit anti-IL-6, dilution 1:30, Santa Cruz Biotechnology, Dallas, TX, USA). After three rinses in fresh PBS, the slides were incubated with horseradish peroxidase-coupled secondary antibody for 40 min at room temperature. Following three additional washes, all specimens were stained with 3,3’-diaminobenzidine (DAB, Dojindo Laboratories, Kumamoto, Japan) substrate. Finally, the sections were rinsed in distilled water and counterstained with Mayer’s hematoxylin, according to the manufacturer’s instructions.

### 4.3. Evaluation of Immunohistochemical Staining

**Figure 3:**
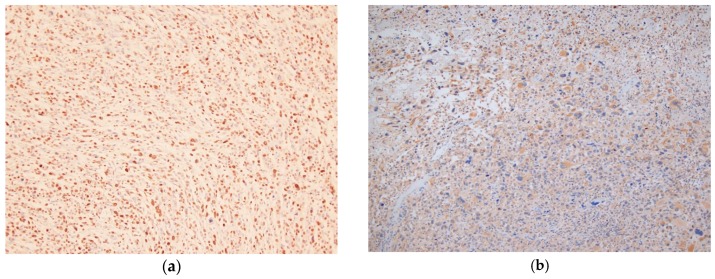
Immunohistochemical staining of IL-6 and IL-6R (**a**) Immunostaining for IL-6 (×100). High expression of IL-6 in tumor tissue. (**b**) Immunostaining for IL-6R (×100). High expression of IL-6R in the tumor tissue.

All sections were assessed by two orthopedic surgeons and one professional technician for pathology (T.I), who were blinded to patient outcomes and all clinicopathologic data. The two orthopedic surgeons (T.N and K.A) are orthopedic oncologists who are officially certified by the Japanese Orthopaedic Association. Expression was assessed semi-quantitatively using two parameters: staining intensity and the percentage of stained tumor cells, as suggested in previous studies [11,14] (Figure 3a,b). The immunohistochemical staining intensity was rated as follows: 1 point, weak intensity; 2 points, intense. Density was rated as follows: 0 point, 0; 1 point, 1–50%; 2 points, 51–75%; and 3 points, 76–100% of positive tumor cells (Figure 3). The eventual score of each specimen was calculated by adding the intensity and density scores. The expression levels of IL-6 and IL-6R were determined as low expression (score ≤ 2) and high expression (score ≥ 3) because the power of the present study for OS and MFS was the highest.

### 4.4. Statistical Analysis

Statistical associations between clinicopathological factors were evaluated using the Mann–Whitney U-test for quantitative data and the chi-squared test and Fisher’s exact test for qualitative data. Correlations between IL-6 expression and/or IL-6R expression in tissues and clinical characteristics were tested using Spearman’s rank-order correlation analysis. 

Survival time was defined as the interval between the date of initial treatment for the primary tumor and the last date on which the patient was documented to be alive or the date of death. Survival curves were constructed using the Kaplan–Meier method. A log-rank test was used to compare the survival of patients exhibiting a high expression of IL-6 or IL-6R versus a low expression. Multivariate analysis was performed using a Cox proportional hazards model, including the significant predictors identified in the univariate analysis as variables. When we validated the power of the study, we defined the 0.05 of the alpha level. We calculated the power from the results of OS and MFS between the patients with both a high expression of IL-6 and IL-6R and those with both a low expression of IL-6 and IL-6R. The factors for multivariate analysis were selected by a stepwise method. A value of *p* < 0.05 was considered significant. All statistical analyses were performed using the EZR graphical user interface (Saitama Medical Center, Jichi Medical University, Saitama, Japan) for R (the R Foundation for Statistical Computing, Vienna, Austria), which is a modified version of R Commander designed to add statistical functions frequently used in biostatistics.

## 5. Conclusions

This study aimed to elucidate the association between IL-6 and IL-6 receptor (IL-6R) expression in tissues and clinical outcomes in patients with STSs. We found that this high expression indicated that the patient had a poor prognosis for OS and MFS.

## Figures and Tables

**Table 1:** The demographics of the 86 patients with soft tissue sarcoma.

Age	Mean (years)	65.6
	Range	10–88
Gender	Male (*n*)	48
	Female (*n*)	38
Tumor depth	Superficial (*n*)	13
	deep (*n*)	73
Tumor size	Mean (cm)	11.1
Grade	High grade (*n*)	61
	Low grade (*n*)	25
Serum IL-6 level	Mean (pg/mL)	30.6

IL-6; Interleukin-6.

**Table 2:** Clinicopathological data of soft tissue sarcoma (STS) patients classified by IL-6 and IL-6R expression in tissues.

Variables		IL6(+), IL6R(+)	IL6(+), IL6R(−) or IL6(−), IL6R(+)	IL6(−), IL6R(−)	*p* Value
		*n* = 14	*n* = 30	*n* = 42	
Age	Mean (years)	71.1	64.5	64.5	0.30
	Median (years)	72.5	66.5	67.5	
Sex	Male (*n*)	4	18	26	0.17
	Female (*n*)	10	12	16	
Depth	Superficial	2	5	6	0.58
	deep (*n*)	12	25	36	
Size	≤10 (cm)	7	21	20	0.29
	>10 (cm)	7	9	22	
	Mean (cm)	11.9	9.48	12.1	0.23
Grade	High grade (*n*)	13	25	23	0.012
	Low grade (*n*)	1	5	19	
CRP level	Mean (mg/dL)	5.3	0.40	0.88	0.000002
Serum IL-6 level	Mean (pg/mL)	87.7	27.6	13.7	0.0039
	Median (pg/mL)	71.5	5.65	5.73	

IL-6; Interleukin-6, IL-6R; Interleukin-6 receptor, (+); high expression, and (−); low expression.

**Table 3:** Univariate cox population hazard analysis for overall survival and metastasis-free survival.

Variables	Parameters	Overall Survival		Metastasis-Free Survival	
		HR (95% CI)	*p* Value	HR (95% CI)	*p* Value
Age	Years	1.073 (1.023–1.125)	0.004	1.033 (1.001–1.066)	0.046
Depth	Superficial	1		1	
	Deep	1.941 (0.451–8.363)	0.37	1.297 (0.454–3.71)	0.63
Size	≤10 cm	1		1	
	>10 cm	1.028 (0.967–1.094)	0.37	0.982 (0.930–1.037)	0.52
Grade	Low grade	1		1	
	High grade	8.682 (1.165–64.71)	0.03	356200000 (0-Inf)	0.99
IL6 and IL6R in tissues	IL6(+), IL6R(+)	5.124 (1.673–15.69)	0.004	6.780 (2.616–17.57)	0.00008
	IL6(+), IL6R(−), or IL6(−), IL6R(+)	1.955 (0.639–5.98)	0.24	2.742 (1.092–6.886)	0.03
	IL6(−), IL6R(−)	1		1	

95% CI; 95% confidential interval, HR; hazard ratio, IL-6; Interleukin-6, IL-6R; Interleukin-6 receptor, (+); high expression, and (−); low expression.

**Table 4:** Multivariate cox population hazard analysis for overall survival.

Variables	Overall Survival	
	HR (95% CI)	*p* Value
Age	1.064 (1.016–1.115)	0.009
Grade high	4.955 (0.634–38.72)	0.13
Low	1	
IL6(+), IL6R(+)	3.537 (1.125–11.12)	0.03
IL6(+), IL6R(−) or IL6(−), IL6R(+)	1.764 (0.559–5.569)	0.33
IL6(−), IL6R(−)	1	

95% CI; 95% confidential interval, HR; hazard ratio, IL-6; Interleukin-6, IL-6R; Interleukin-6 receptor, (+); high expression, and (−); low expression.

**Table 5:** Multivariate cox population hazard analysis for metastasis-free survival.

Variables	Metastasis-Free Survival	
	HR (95% CI)	*p* Value
Age	1.030 (0.998–1.064)	0.07
IL6(+), IL6R(+)	6.219 (2.389–16.19)	0.0002
IL6(+), IL6R(−) or IL6(−), IL6R(+)	2.909 (1.153–7.340)	0.02
IL6(−), IL6R(−)	1	

95% CI; 95% confidential interval, HR; hazard ratio, IL-6; Interleukin-6, IL-6R; Interleukin-6 receptor, (+); high expression, and (−); low expression.
